# Artemisinin combination therapies price disparity between government and private health sectors and its implication on antimalarial drug consumption pattern in Morogoro Urban District, Tanzania

**DOI:** 10.1186/1756-0500-5-165

**Published:** 2012-03-28

**Authors:** Allen Lewis Malisa, Deodatus Kiriba

**Affiliations:** 1 Department of Biological Sciences, Faculty of Science, Sokoine University of Agriculture, Box 3038, Morogoro, Tanzaia

## Abstract

**Background:**

Universal access to effective treatments is a goal of the Roll Back Malaria Partnership. However, despite official commitments and substantial increases in financing, this objective remains elusive, as development assistance continue to be routed largely through government channels, leaving the much needed highly effective treatments inaccessible or unaffordable to those seeking services in the private sector.

**Methods:**

To quantify the effect of price disparity between the government and private health systems, this study have audited 92 government and private Drug Selling Units (DSUs) in Morogoro urban district in Tanzania to determine the levels, trend and consumption pattern of antimalarial drugs in the two health systems. A combination of observation, interviews and questionnaire administered to the service providers of the randomly selected DSUs were used to collect data.

**Results:**

ALU was the most selling antimalarial drug in the government health system at a subsidized price of 300 TShs (0.18 US$). By contrast, ALU that was available in the private sector (coartem) was being sold at a price of about 10,000 TShs (5.9 US$), the price that was by far unaffordable, prompting people to resort to cheap but failed drugs. As a result, metakelfin (the phased out drug) was the most selling drug in the private health system at a price ranging from 500 to 2,000 TShs (0.29–1.18 US$).

**Conclusions:**

In order for the prompt diagnosis and treatment with effective drugs intervention to have big impact on malaria in mostly low socioeconomic malaria-endemic areas of Africa, inequities in affordability and access to effective treatment must be eliminated. For this to be ensued, subsidized drugs should be made available in both government and private health sectors to promote a universal access to effective safe and affordable life saving antimalarial drugs.

## Background

Prompt treatment with effective antimalarial drugs is among major strategies for control of the life-threatening malaria disease. However, the success of this strategy faces daunting challenges of spread of drug resistance and impediment of access to effective treatment for malaria [1]. Development assistance has been routed largely through government channels, whereas affected individuals seek treatment mostly through the private sector and the new artemisinin-based combination treatments (ACTs), recommended by WHO for uncomplicated falciparum malaria, are too expensive for many people who seek treatment in the private sector [1]. Although it is widely recognized that, the government health sector plays a central role in the delivery of key curative and preventive interventions for malaria in most African countries, government sector alone do not guarantee sufficient levels of access in malaria endemic countries [2]. Evidence from surveys conducted between 2007 and 2008 in 11 countries across Africa indicates that despite large increase in the number of antimalarial treatments supplied through the government health sector, only 15% of children with fever were treated with ACTs [3].

Treatment of malaria in Tanzania is typically guided by official recommendations from the Ministry of Health and Social Welfare (MOHSW) regarding drugs of choice for various situations. “First-line” treatment refers to the drug officially recommended as the drug of first choice for the treatment of uncomplicated malaria. “Second-line” treatment refers to the drug officially recommended as an alternative primarily to be used for treatment of patients in whom the first-line treatment failed to clear the infection and other select patients (such as those who are hypersensitive to the first-line treatment). “Third-line” treatment typically refers to the drug recommended for severely ill patients (a rescue drug). In practice, few treatment failures are recognized and patients are often moved directly from first to third-line treatment, consequently, little second-line drug is used compared to the first-line drug. Tanzania changed her antimalarial policy for the first time in 2001, when CQ first line was replaced by SP as an interim first line policy, while evaluating a long term solution. In 2006, SP was replaced with Artemether + Lumefantrine (ALU) combination therapy as the newly adopted first line antimalarial option for Tanzania [4]. At an ex-manufacturer price of up to 11 US dollars (USD) per adult treatment course, ACTs are 20–40 times more expensive than conventional antimalarial medicines [1,5]. The adoption of ACTs by malaria endemic countries has been made possible largely because of an increased financial support provided by the global fund to fight AIDS, Tuberculosis and Malaria (GFATM). Newer initiatives to support malaria control such as the US president’s Malaria initiative (PMI) and the World Bank Booster Program which were launched in 2005 are additional sources of support for this change, but until now, their financial disbursements have been considerably less than those of the GFATM [6]. The advent of the Affordable Medicines Facility for malaria (AMFm) in 2010 however, is destined to supersede initial schemes providing opportunity for up scaling to make ACT affordable and accessible [2]. AMFm is a financing mechanism designed to subsidize the most effective anti-malaria drugs, ACTs; Tanzania being among the 10 countries invited to pilot a first phase of this subsidy as from mid 2010. Through co-payment and subsidy; first-line buyers, including in-country private sector wholesalers, hospitals and NGOs, will pass on the benefit to patients who will pay a price of $0.20–0.50 for ACTs, a price which is comparable to what they are currently paying for less-effective alternatives.

This unique partnership is using innovative technology to reduce the cost of ACTs which are still beyond the reach of the world’s poorest people, thereby making these life-saving therapies more accessible to people in the developing world.

The supply of subsidized ACTs in Tanzania is channeled through the government health sector at an affordable price of 300 TShs (0.18 US$). Until recently, ALU were only available in government health facilities. Recently, however, few drug selling outlets branded as Accredited Drug Distribution Outlets (ADDO) also known as “duka la dawa baridi” are being evaluated and will be licensed to sell the subsidized ALU, but it is still under pilot study. Review of available literature reveal that drug shops and pharmacies are often preferred to health facilities as a first treatment action [2]. Some of attributes making retailers more preferred include their tendency to be more accessible, quick services, have longer and more flexible opening hours, are willing to negotiate charges and offer credits, are more polite and friendlier and are generally perceived as being cheaper. Not withstanding, government health facilities experience frequent stockouts leaving no option to patient other than private drug shops and outlets. Since the ACTs that are available in the private health sector are not subsidized, their prices ranges from 10,000 to 15, 000 TShs (5.9–8.8 US$) which is approximately 30–50 times more expensive than those sold in the government health sector. These prices are by far unaffordable by an average Tanzanian citizen, prompting them to resort to using inexpensive but failing drugs such as, CQ, AQ or SP. Alternatively, some patients may use artemisinin monotherapies, which are generally cheaper than ACTs but could accelerate the development of parasite resistance as has been suggested elsewhere [7,8]. Although WHO discourages the use of artemisinins in monotheapy form, in the new era of ACTs, there is a concern that medicine sellers will continue to sell artemisinin derivatives alone (monotherapies), potentially jeopardizing ACT efficacy in the long-term [9]. Despite this concern, adequate data on levels/trends of use and prices of ACTs in both the private and government health systems continue to be lacking. The present study audited both government and private DSUs in Morogoro urban district to assess the implication of price on the availability and use pattern of ACTs in the private sector.

## Methods

### Study area

The study was conducted in Morogoro Urban District between January and April 2010, specifically involving government and private health facilities (hospital, health centre and dispensary), pharmacies and drug stores (“duka la dawa baridi”) located at different parts of the District. For the purpose of this study, government and private health facilities, pharmacies and drug selling stores/shops and outlets are hereby referred to as drug selling units (DSU), and hence two categories shall be referred here as the government drug selling unit (GDSU) and private drug selling unit (PDSU), respectively. The study area spans a total of 260 Km^2^, which is about 0.4% of 72,939 Km^2^ total area of Morogoro Region. *Plasmodium falciparum* malaria transmission in the study area is intense (with an estimated entomological inoculation rate of 367 infectious bites per person per year [10] and perennial with some seasonal fluctuation (although recent studies indicate decrease in malaria transmission in endemic areas but new entomological inoculation rate for the studied area has not been determined).

According to the National population census of 2002, Morogoro municipal had a population of 235,000 people and an average population growth rate of 4.6% [11]. Morogoro Urban has diverse ethnicity in which Luguru and Pogoro being the majority are located across the municipality, while other ethnic groups who identifies themselves as Wachaga, Wasukuma, Wapare, Wanyakyusa, Wahaya, Wahehe, Wasambaa, Wangoni and many others are also found in substantial numbers. Major religions are Christianity and Islam. Major economic activities in the study area include trade (both retail and wholesale), commercial and subsistence urban farming and employment in both government and private sector. The area is served by 3 government hospitals (one of them is the Morogoro regional referral hospital), 9 health centres (4 government and 5 private), 32 dispensaries (20 government and 12 private), 14 private pharmacies and 118 private medical stores (duka la dawa baridi) (Table 1).

**Table 1:** Number and categories of drug selling units (government and private) serving the Morogoro Urban District

**S/N**	**Drug selling unit category**	**Government**	**Private**
1	Hospital	3	0
2	Health centre	4	5
3	Dispensary	20	12
4	Pharmacy	0	14
5	Medical store (duka la dawa baridi)	0	118
	**Total**	**27**	**149**

The study involved physical visit to the ascribed drug selling units located in Morogoro Municipality to collect data on levels and trends in price, availability and consumption of antimalarials in Morogoro urban. A total of 92 DSUs (14 GDSUs and 78 PDSUs) were randomly visited and questionnaire interviews administered to the service providers.

### Study method and data collection

The study employed stratified random sampling to include both GDSUs and PDSUs and for each, to include all categories of DSU where applicable (hospital, health center, dispensary, pharmacy and drug shop or “duka la dawa baridi”). The inclusion criteria were; any unit within the study area stocking and selling antimalarial drugs and willingness to consent and participate in the study. Sample size of 92 DSUs was calculated using sample size calculator [12] from 176 DSUs serving the region (Table 1) at a confidence interval and level of 95% and 7, respectively. A combination of observation, interviews and questionnaire administered to the service providers of the DSUs, were used to collect data. Quantitative data was collected using interviewer-administered questionnaires that were administered to all health providers of the randomly selected DSUs including 14 government (2 hospitals, 2 health centres and 10 dispensaries) and 78 private (3 health centres, 6 dispensaries, 7 pharmacies and 62 medical stores). The questionnaires focused on research questions to assess the type and rate of antimalarial consumption between GDSUs and PDSUs and price and availability of ACTs between the two sectors. Few social-demographic data including age, religion, sex and education was included in the questionnaires. An interview guide comprising of 17 open-ended questions in Kiswahili was pre-tested on a sample of six drug store service providers who were drawn from the original sampling frame. Their responses, which were not included in the final data analysis, were reviewed and the questions refined in light of the pre-test. Detailed interviews were then completed with 92 DSUs providers.

### Data analysis

The quantitative data from primary source was edited and coded. The hand sheets were prepared and data entered into a computer using Microsoft Excel. Data analysis was done using Statistical Package for Social Science (SPSS) software and results generated in frequency tables. Microsoft Excel was used to generate histograms.

### Ethics approval

Institutional clearance of the research was sought from Sokoine University and consent was obtained from participants before interview.

## Results

Of the 92 respondents, 50% were aged between 15 and 30 years, 30.5% were between 31 and 45 years of age, 15.2% were between 46 and 60 years of age and the remaining 4.3% were above 60 years old. In terms of sex, 67.4% were females while 32.6% were males. The specialty of respondents indicated that out of 92 respondents, majority (52.2%) were nurses, 19.6% were doctors, 15.2% were pharmacists and 13.0% were drug sellers (Table 2).

**Table 2:** Social demographic information of the respondents (N = 92)

**Variable**	**Response**	**Number (%)**
**Sex**	Male	30 (32.6)
	Female	62 (67.4)
**Age**	15–30	46 (50.0)
	31–45	28 (30.5)
	46–60	14 (15.2)
	>60	4 (4.3)
**Specialty**	Nurse	48 (52.2)
	Doctor	18 (19.6)
	Pharmacist	14 (15.2)
	Drug seller	12 (13.0)
**Education level**	Primary education	10 (10.9)
	Secondary education	48 (52.2)
	Advanced level education	4 (4.3)
	College/University education	30 (32.6)
**Marital status**	Married	52 (56.5)
	Single	36 (39.1)
	Divorced	4 (4.3)
**Religion**	Muslim	16 (17.4)
	Christian	76 (82.6)

The education levels of the respondents showed that 10.9% had primary education, 52.2% had secondary education, 4.3% had advanced education and the remaining 32.6% had college/University education. Marital status of respondents suggested that 56.5% were married, 39.1% were single while the remaining 4.3% were divorced. Majority (82.6%) of the respondents were Christians and the remaining 17.4% were Muslims (Table 2).

### Levels of awareness of first line drug for treatment of malaria in Tanzania

**Figure 1:**
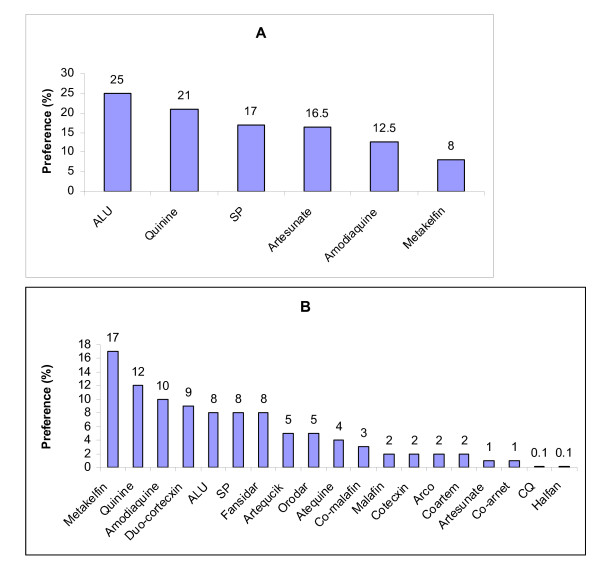
**Customers’ preference for antimalarial drugs in Morogoro urban.** A is GDSUs and B is PDSUs.

A total of 92 DSUs in Morogoro urban, of which 78 were private and 14 government, were surveyed. Eighteen (18) different antimalarial drugs were being sold in the 92 visited DSUs in Morogoro municipal (Figure 1). The PDSUs were the most diverse, as they were selling all the eighteen (18) varieties of antimalarials available at Morogoro municipal (Figure 1 B), while the GDSUs were less diverse, selling only six (6) varieties, which were a subset of those sold in PDUs (Figure 1 A). The respondents to the questionnaire administered to the visited DSUs were the service providers. The respondents were asked whether they know the nationally approved first line antimalarial treatment. As expected, all the service providers of the GDSUs indicated they know, and correctly mentioned ALU (Artemether + Lumefantrine) is the approved drug. Since the GDSUs were composed of health facilities only (hospitals, health centres and dispensaries), drug dispensing were done only after prescription from medical doctor or a clinical officer, and hence in the present study, detailed assessment of awareness of the national antimalarial policy focused mainly to PDSUs (Table 3).

**Table 3:** Awareness of the national antimalarial policy and factors influencing antimalarial consumption in the private health sector (N = 78)

**Variable**	**Response**	**Number (%)**
**Awareness of 1**^**st**^**line drug**	Aware	76 (97.5)
	Unaware	2 (2.5)
**Name of 1**^**st**^**line drug**	ALU	70 (90.0)
	Coartem	6 (7.0)
	Metakelfin	2 (3.0)
**Buying without prescription/ consultation**	Majority	20 (25.0)
	Moderate	27 (35.0)
	Few	31 (40.0)
**Factors influencing drug choice**	Price and efficacy	68 (87.0)
	Drug popularity	8(10.0)
	Others	2 (3.0)

Out of 78 respondents from PDSUs, 97.5% indicated to know the first line drug, while only 2.5% respondents indicated to be unaware. When required to mention the approved first line national antimalaria treatment drug, 97.4% of the respondents who indicated to know it, mentioned correctly ALU (Artemether + Lumefantrine-92%) or Coartem® (Artmether + Lumefantrine-8%) while 2.6% mentioned wrongly Metakelfin® (Sulfamethoxypyrazine + Pyrimethamne). The respondents were also requested to comment on proportions of customers visiting the drug selling units, either with prescription from medical doctor or to seek expert advice from PDSUs’ service providers, as compared to those who visit to purchase without doctor’s prescription or the need for expert advice. Sixty percent (60%) indicated majority to moderate, while 40% indicated few numbers of customers purchase without prescription or consultation.

The respondents were also asked to list the most selling (preferred) drugs for malaria treatment in their order of preference. The order of preference and the number of times the drug was mentioned by different respondents were harmonized into a percentage score (Figure 1 B). Surprisingly, metakelfin was the most selling drug with a score of 17%, followed by quinine (12%), amodiaquine (10%) and duo-cotecxin (9%). ALU, the nationally recommended first line antimalarial drug ranked the fifth similar to fansidar and SP each with a sore of 8%. Orodar and artequick ranked the sixth, each scoring 5%, artequine scored 4% and ranked the seventh and co-malafin scored 3% and ranked eighth. Malafin, Arco, cotecxin and Coartem scoring 2% each, ranked ninth, artesunate and co-arinate each scoring 1% ranked tenth and CQ and halfan scored 0.1 each and ranked eleventh. It was also observed that, the number of PDSUs selling coartem (the ALU substitute in the private sector) were concentrated in the urban centre and as you move away toward the peri urban areas, the number decreased gradually.

However, in the GDSUs, as expected, ALU was the most selling drug scoring 25%, quinine the second scoring 21%, SP the third scoring 17%, artesunate the fourth scoring 15.5%, amodiaquine the fifth scoring 12.5% and metakelfin the sixth scoring 8% (Figure 1 A).

When the respondents were asked to give their opinion on what determines consumer choices, 87% mentioned price and efficacy of the drug, 10% mentioned popularity of the drug and 3% mentioned other factors. When required to specify the other factors determining buyer’s choice they mentioned absence of adverse drug reactions and the dosage regimen. Drugs with shorter period of dosage completion (such as those taken only once, e. g. metakelfin) are more preferred.

The subsidized artemether + lumefantrine (ALU) were meant to be distributed and hence sold solely in the government health system and therefore, for easier diferentiation from other drug preparations they were ordered from a single manufacturer, Novartis, Switzerland in a special and unique blister preparation. To confirm the absence of subsidized ALU from PDSUs, we asked the respondents whether their units were selling unauthorized ALU or not. Of the 78 PDSUs studied, 31% were selling unauthorized ALU while 69% were not. Of the 24 units found selling unauthorized ALU, 50.0% of them were selling it at large to moderate scale (quantities) while the other 50.0% were selling at a low scale (quantities).

### Antimalarial drugs prices

**Figure 2:**
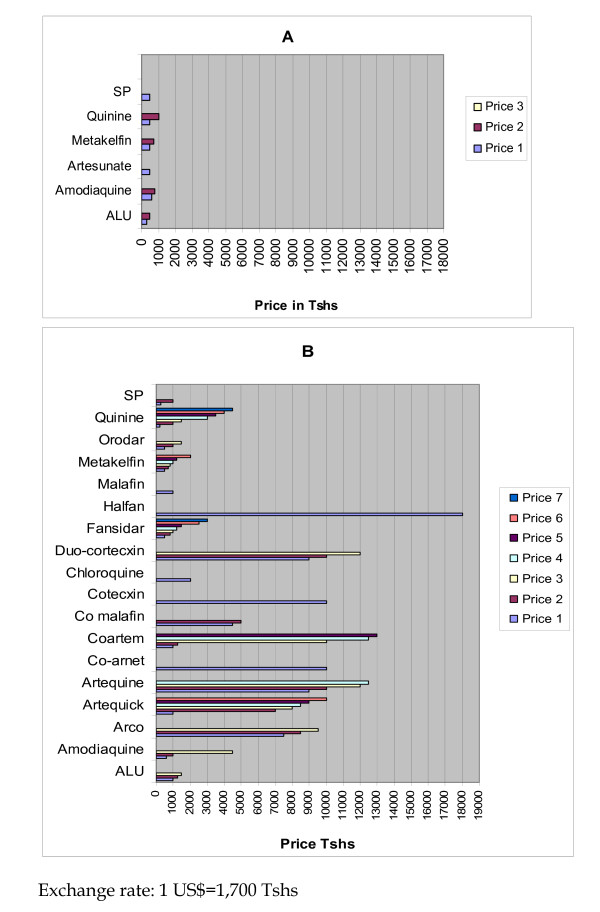
**Price range of antimalarial drugs in Morogoro urban**. A is for government and B is for private health sector. Note that some drugs like fansidar had 7 different prices across the surveyed DSUs in the study area.

The results of the survey of antimalarial drugs prices in Morogoro municipal are shown in Figure 2. The data shows a big difference in prices between the PDSUs and GDSUs (See Figure 2). The GDSUs sold only six varieties of antimalarial drugs, which were a subset of the eighteen (18) varieties found in the PDSUs at an affordable price range between 300 and 1,000 TShs (0.18–0.59 US$). To the contrary, the PDSUs in Morogoro urban district, beside their diversity in terms of stocking and selling 18 varieties of antimalarial drugs, it revealed an amazing diverse list of different prices for a single variety of drug (Figure 2 B). Quinine and fansidar, for instance, each had 7 different prices, with prices ranging from 200 to 4,500 TShs (0.12–2.64 US$) and 500–3,000 TShs (0.29–1.76 US$), respectively. The spectrum of different prices for the same antimalarial drug variety compelled the researcher to conduct a verbal interview with the PDSUs service providers. When providers were asked, what was the reason for one antimalarial variety to have up to seven different prices?; this is what they had to say: “multiple prices for the same drug is a result of two things; one, free market trade which allow the seller to fix a price of choice, but, two, lack of strict enforcement of importation regulations leading to porous national boundaries allowing the infiltration of all possible drug generics from across the world that includes quality and low quality alternatives, hence widely divergent prices”. In general, there was a big difference in terms of antimalarial drug prices between GDSUs and PDSUs. The recommended first line antimalarial treatment in Tanzania ALU for instance, had a price of 300 TShs (0.18 US$) in the GDSUs compared to its substitute Coartem in PDSUs sold at 13,000 TShs (7.7 US$) p < 0.0001, and this difference was highly statistically significant.

## Discussion and conclusions

This study has generated data on levels, trends of use and prices of ACTs in both the PDSUs and GDSUs in Morogoro Urban District in Tanzania. The information generated is useful in several ways; firstly it provides the understanding of the level of uptake of the national antimalarial guideline, giving an indication of the fraction of the population that is accessible to effective, safe and life saving drugs (ACTs); secondly, it provides field data on the effect of high cost in, not only limiting access/affordability of ACTs, but also subjecting those who are in need of them to resort to cheap but failing alternative drugs such as CQ and SP generics. The study also reports the findings of competence and awareness of the service providers of the DSUs in both government (GDSUs) and private (PDSUs) in the study area. The World Health Organisation (WHO) first endorsed ACTs for the treatment of malaria in 2004 and recommended a switch to ACTs as the first line malaria treatment in 2005 [13]. Subsequent to the endorsement, in January 2006, WHO made a strong appeal to pharmaceutical companies, National Drug Regulatory Authorities and international funding and procurement agencies to manufacture, procure and promote ACTs as the best standard of care for malaria treatment and called for an end to the deployment of artemisinin monotherapies for the treatment of uncomplicated malaria (a practice especially common in the private sector) to prevent the development of resistance to artemisinins [5].

The study found a very distinct consumption pattern of antimalarial treatments between the government health system and the private health system. In the government health system ALU (a nationally recommended first line treatment) was the most selling drug followed by quinine. On the contrast, in the private system, metakelfin (sulfamethoxypyrazine + pyrimethamine) (a failed drug that was replaced by ALU in the 2006 antimalarial policy review) was the most selling drug followed by quinine as in the government sector. However, this finding is not surprising, given the price of coartem (a substitute of ALU in PDSUs) which goes up to 13,000 TShs (7.7 US$) per adult dose, a price that is more than 40 times the price of ALU (300 TShs or 0.18 US$) in the GDSUs. This finding is consistent with recent baseline study of the private market for antimalarials in Muheza town reporting SP to be the most consumed drug with consumption rate of 74%, followed by AQ (13%), quinine (11%) and ACT (2%) [14].

Since policy change from SP first line to ALU in 2006, the supply of subsidized ALU in Tanzania has been solely through government system until recently, when Ministry of Health and Social Welfare (MOHSW) in collaboration with Management Sciences for Health (MSH) proposed and launched a pilot program to improve private drug stores popularly known as “duka la dawa baridi” and promote them to improved drug shops known as Accredited Drug Distribution Outlets (ADDO) popularly known as “duka la dawa muhimu”, which will be mandated to supply ALU in private sector. Accordingly, most of the 118 drug stores available in Morogoro Urban District will be improved but in phases. To date, only 35 (30%) drug stores have been promoted and recent informal survey (data not shown) indicates some of them continue to sell the subsidized ALU at a higher price ranging from 1,000–3,000 TShs (0.59–1.76 US$) instead of the approved 300 TShs (0.18 US$). Besides ALU being very unpopular (ranked the sixth in the list of most selling antimalarial drugs in this study) in the PDSUs, high price of other ACTs (Coartem-an ALU substitute, Duo-cotecxin, Artequine, Artequick and Arco) has almost driven people to resort to inexpensive but failed drugs including SP, CQ, malafin, orodar, fansidar etc. This finding is similar to reports elsewhere [15] suggesting that unfortunately because of their high prices, ACTs are not widely accessible to the people who most need them and many of whom resort to inexpensive but failing drugs such as CQ. In the present study the effect of price on the accessibility/affordability of highly efficacious but very costly ACTs were much more marked in the peri-urban where most of ACTs were not even stocked by the DSUs serving the area, because, due to their high cost people could not afford buying them. This could be explained by difference in socioeconomic status whereby those residing in the urban centre are wealthier and hence can afford better but more costly drugs than their peri-urban counterparts. Similar findings had been reported previously by [16], who conducted a community-based survey in Nigeria and demonstrated that those earning greater than N3000/day were twice as likely to use “appropriate” drugs (defined as drugs to which resistance had not developed – Fansidar mainly) compared to those earning less than N3000/day who were more likely to use CQ, despite resistance.

Among the concerns of the government regarding the supply of ALU in the government sector alone, was the fear that, ALU would find its way and leak to the private sector. To address this problem, ALU which is supplied through the government system were uniquely packed in single dose blisters that were very different from the Coartem (ALU-alternative that was available in the private sector). Notwithstanding, ALU leaked out of government health sector, and in this study, 30% of surveyed PDSUs in Morogoro urban district were selling ALU illegally at a slightly higher price ranging from 1,000 to 1,500 TShs (0.59–0.88 US$) compared to 300 TShs (0.18 US$) in the government sector. It is hoped that AMFm initiative with its subsidy and co-funding approach, if carefully monitored for intended services to reach recipients, will most likely help to save lives and reduce the use of less-effective malaria treatments by increasing access to ACTs and displacing artemisinin monotherapies from the market [17].

In the new era of ACTs, one of the major concerns has been the possibility that medicine sellers may continue selling artemisinin derivatives in monotherapy form, potentially jeopardizing ACT efficacy in the long term [9]. In the present study, the government sector was found selling artemether while the private sector was selling artemether and cotecxin, both of which are artemisinin based monotherapies. While the use of artemether may be explained by the fact that it may likely be used in severe cases of malaria (although the practice need to be discouraged), the use of cotecxin and artemether in the private sector underscores the need for the education and other mechanisms to discourage the use of artemisinin based monotherapies to avoid artemisinin resistance; hence AMFm initiative is expected to play pivotal role on this.

The knowledge of dispensers regarding the recommended first line malaria treatment was encouraging, although 4% of the 92 dispensers interviewed, did not know that ALU is the nationally recommended treatment for uncomplicated malaria. Of the 4%, half of them genuinely confessed to be unaware, while the other half claimed to know the drug but when requested to name it, mentioned metakelfin. This proportion may seem to be small but is very significant, given the number of people they are serving, raising concerns surrounding appropriateness of the drugs and information that these people provide to their customers. The problem of unskilled medicine sellers is well documented and widespread [18-20], and extensive review of medicine sellers in eastern African countries of Kenya and Uganda suggest that, their levels of education vary greatly, but most have little or no formal training in medicine or pharmacy [21]. Accordingly, drug shop/outlet/store staff may be untrained, or trained as medical assistants or nurses, and even where the official owner or licensee has health-related qualifications, outlets are often staffed by less qualified assistants. In the present study, majority (52%) of drug dispensers were nurses, 20% were medical doctors/clinical officers, 13% were just drug sellers while only 15% were pharmacists. Of the drug sellers, 85% had primary education while only 15% had secondary education. Clearly, to achieve a well-regulated and properly operated private antimalarial selling units, proper licensing accompanied by close supervision and monitoring is mandatory. Ideally, all antimalarial selling units/outlets should be manned by a skilled drug dispenser and this should be a pre-requisite for obtaining a license.

In conclusion, this study has quantified the influence of high price on the levels, trend and consumption pattern of antimalarial drugs in Morogoro urban district. The highly efficacious national first line antimalarial drug ALU was only accessible and affordable for those who sought medical service through government health system at a subsidized price of 300 TShs (0.18 US$) but its substitute in the private health system (coartem) which was being sold at a price of about 10,000 TShs (5.9 US$) per adult dose, was by far unaffordable. Other ACTs were also sold at similar or close price forcing people to resort to either inexpensive but failed drugs such as metakelfin, SP, CQ, malafin, orodar, fansidar or relatively less expensive artemisinin monotherapies such as cotecxin and artemether. More than 13% of surveyed DSUs were manned by unskilled personnel. Although this study was done in Mrogoro urban area, the experience described is of greater relevance and the results likely reflect the situation in the whole country although a further large scale study including the rural area will give a more representative picture of the entire country. The study recommends that; since the private sector still plays a major role in antimalarial drug delivery, and the current framework of antimalarial drug delivery through government sector alone has failed to provide sufficient ACT coverage to make major impact on malaria, it is inevitable that for successful malaria control effort, subsidized ACTs should be delivered through both government and private health system to promote access and affordability to all who are in need of them. Again, the current Ministry of Health and Social Welfare idea of using selected improved private drug shops popularly known as ADDO will most likely not achieve the highly needed wider delivery of the much needed ACTs in the country.

## Abbreviations

ACT = Artemisinin combination therapy; ALU = Artemether + lumefantrine; ADDO = Accredited drug distribution outlets; CQ = Chloroquine; DSU = Drug selling unit; GDSU = Government drug selling unit; PDSU = Private drug selling unit; MSH = Management sciences for health; MOHSW = Ministry of health and social welfare; SP = Sulphadoxine and Pyrimethamine; WHO = World Health Organization.

## Competing interests

The authors declare that they have no competing interest.

## Authors’ contribution

Both authors contributed to the study design. ALM participated in data collection and analysis and drafting of the manuscript. DK participated in data collection and analysis and critical review of the manuscript for intellectual content. Both authors read and approved the final manuscript.
